# (Cost-)effectiveness of a personalized multidisciplinary eHealth intervention for knee arthroplasty patients to enhance return to activities of daily life, work and sports – rationale and protocol of the multicentre ACTIVE randomized controlled trial

**DOI:** 10.1186/s12891-023-06236-w

**Published:** 2023-03-04

**Authors:** A. Carlien Straat, Jantine M. Maarleveld, Denise J. M. Smit, Lara Visch, Gerben Hulsegge, Judith A. F. Huirne, J. M. van Dongen, Rutger C. van Geenen, Gino M. M. J. Kerkhoffs, Johannes R. Anema, Pieter Coenen, P. Paul F. M. Kuijer

**Affiliations:** 1 Amsterdam UMC, Department of Public and Occupational Health, Van Der Boechorststraat 7, Amsterdam, 1081 BT the Netherlands; 2 Amsterdam Public Health Research Institute, Societal Participation and Health, Amsterdam, the Netherlands; 3 Amsterdam Movement Sciences Research Institute, Musculoskeletal Health, Sports, Amsterdam, the Netherlands; 4 Centre for Nutrition, Prevention and Health Services, National Institute for Public Health and the Environment, Bilthoven, the Netherlands; 5 Department of Research, Sint Maartenskliniek, Nijmegen, The Netherlands; 6 Sustainable Productivity & Employability, Netherlands Organization for Applied Scientific Research TNO, Leiden, the Netherlands; 7 Department of Obstetrics and Gynecology, Amsterdam UMC, Location VU Medical Center, Amsterdam, The Netherlands; 8 Amsterdam Reproduction and Development Research Institute, Amsterdam, the Netherlands; 9 Department of Health Sciences, Faculty of Science, Vrije Universiteit Amsterdam, Amsterdam Movement Sciences Research Institute, Amsterdam, The Netherlands; 10 Department of Health Sciences, Faculty of Science, Vrije Universiteit Amsterdam, Amsterdam Public Health Research Institute, Amsterdam, The Netherlands; 11 Department of Orthopaedic Surgery, FORCE (Foundation for Orthopaedic Research Care Education), Amphia Hospital, Breda, The Netherlands; 12 Department of Orthopaedic Surgery and Sports Medicine, Amsterdam Movement Sciences, Amsterdam University Medical Center, Location Academic Medical Center, University of Amsterdam, Amsterdam, The Netherlands; 13 Amsterdam Public Health Research Institute, Societal Participation and Health, Quality of Care, Amsterdam, the Netherlands

**Keywords:** Arthroplasty, Replacement, Knee, Cost–benefit analysis, Quality of life, Return to work, Fitness trackers, Mobile applications, Telemedicine, Delivery of health care

## Abstract

**Background:**

With the worldwide rising obesity epidemic and the aging population, it is essential to deliver (cost-)effective care that results in enhanced societal participation among knee arthroplasty patients. The purpose of this study is to describe the development, content, and protocol of our (cost-)effectiveness study that assesses a perioperative integrated care program, including a personalized eHealth app, for knee arthroplasty patients aimed to enhance societal participation post-surgery compared to care as usual.

**Methods:**

The intervention will be tested in a multicentre randomized controlled trial with eleven participating Dutch medical centers (i.e., hospitals and clinics). Working patients on the waiting-list for a total- or unicompartmental knee arthroplasty with the intention to return to work after surgery will be included. After pre-stratification on medical centre with or without eHealth as usual care, operation procedure (total- or unicompartmental knee arthroplasty) and recovery expectations regarding return to work, randomization will take place at the patient-level. A minimum of 138 patients will be included in both the intervention and control group, 276 in total. The control group will receive usual care. On top of care as usual, patients in the intervention group will receive an intervention consisting of three components: 1) a personalized eHealth intervention called ikHerstel (‘I Recover’) including an activity tracker, 2) goal setting using goal attainment scaling to improve rehabilitation and 3) a referral to a case-manager. Our main outcome is quality of life, based on patient-reported physical functioning (using PROMIS-PF). (Cost-)effectiveness will be assessed from a healthcare and societal perspective. Data collection has been started in 2020 and is expected to finish in 2024.

**Discussion:**

Improving societal participation for knee arthroplasty is relevant for patients, health care providers, employers and society. This multicentre randomized controlled trial will evaluate the (cost-)effectiveness of a personalized integrated care program for knee arthroplasty patients, consisting of effective intervention components based on previous studies, compared to care as usual.

**Trial registration:**

Trialsearch.who.int; reference no. NL8525, reference date version 1: 14–04-2020.

**Supplementary Information:**

The online version contains supplementary material available at 10.1186/s12891-023-06236-w.

## Introduction

Knee osteoarthritis is the most prevalent joint disease among adults in many developed countries [1–4]. In the coming years, the ageing population and the obesity epidemic will further increase the number of osteoarthritis patients. Osteoarthritis is one of the leading causes of pain and disability worldwide [5]. This burden is particularly high for those receiving knee arthroplasty. Partial or unicompartmental knee arthroplasty and total knee arthroplasty are well established treatment options for end-stage knee osteoarthritis [6]. Many developed countries expect a high increase in the projected burden of knee arthroplasty. For example, in the Netherlands, an increase of 279% is expected between 2005 and 2030, and in Australia of 276% between 2013 and 2030 [7–9]. Importantly, in 2030, half of the knee arthroplasty patients are expected to be of working age [10]. These patients are often socially active and tend to have high expectations regarding return to daily life activities, including work and sports, after surgery [11, 12].

The standard clinical care of knee arthroplasty patients is not primarily aimed at societal participation, but is mostly aimed at classic clinical outcomes, such as pain relief and knee function. Once the patient has been discharged after surgery, often within one or two days, there is limited guidance and monitoring regarding recovery of societal participation in work and sports [13, 14]. We recently showed that recommendations for return to daily life activities, including work and sport, are often missing and vary considerably in Dutch hospitals and clinics, and that more uniformity across health care providers regarding recommendations for postoperative societal participation is needed [15]. Moreover, return to work [16, 17] and return to sport [18] rates are lower than might be expected based on the good clinical outcomes of knee arthroplasty. Three out of ten Dutch knee arthroplasty patients do not return to work at all, and only 50% of the patients accomplish return to work within three months [16, 17]. A range of 36 to 89% (total knee arthroplasty) and 75 to 100% (unicompartmental knee arthroplasty) of patients are able to return to sport after surgery, which takes those who return to sports on average 12–13 weeks [18]. As societal participation is an important predictor of recovery, good health and quality of life [19, 20], the low return to work and return to sport rates potentially contribute to poor general- and mental health. In addition, these low rates of return to work and to activities of daily life also result in a large economic burden for society due to long sick leave periods and hence high costs from paid and unpaid productivity losses [21].

Considering the above, improving perioperative care, especially for younger knee arthroplasty patients, with a primary focus on societal participation, could contribute to a faster return to activities of daily life, work and sports, thereby improving quality of life and reducing healthcare and societal costs. This paper describes the development, content, and protocol of the multicentre ACTIVE (enhAncing soCietal parTicipation with an IndiVidualized integratEd care program) trial. In this study we will test a new transmural integrated care intervention, in which we combine previously identified effective elements by our research group (i.e. eHealth, goal setting using goal attainment scaling and a referral to a case-manager) to increase societal participation of knee arthroplasty patients [14]. We will examine whether adding this integrated care program to usual care is (cost-)effective as compared to the care as usual only for the resumption of daily life activities and improving quality of life in working age knee arthroplasty patients.

## Methods

### Trial design

The ACTIVE trial is a multicentre, parallel randomized controlled trial with an effectiveness and economic evaluation. Design and reporting of this study will be done in accordance to the CONSORT statement [22] and the CHEERS statement [23], while this protocol is reported according to the SPIRIT guidelines and checklist (Appendix 1) [24]. This study will be conducted according to the principles of the Declaration of Helsinki, and in accordance with the Dutch Medical Research Involving Human Subjects Act (WMO) and with the Dutch Personal Data Protection Act. This study has been approved by the Medical Ethical Committee of Amsterdam UMC, location VUmc, under registration number NL67692.029.18. It has been registered at the Netherlands Trial Register with reference no. NL8525 (reference date version 1: 14–04-2020), but, due to the probation of this website, can now be found at the International Clinical Trials Registry Platform from the World Health Organization (trialsearch.who.int; reference no. NL8525). Substantial changes to the protocol will be registered in the trial register (of which an audit trail will be maintained) and will be reported to the Medical Ethical Committee. Patients allocated to the intervention group will, on top of care as usual, receive a transmural integrated care program, i.e. aimed at the needs of the patient and developed by a multidisciplinary team, to support them in return to daily life activities, including work and sports. The intervention will consist of three components including the personalized ikHerstel app – (which literally translates to the “I Recover” app) and will last until full resumption of daily life activities is achieved (with a maximum of 12 months). Our intervention will be compared to care as usual.

### Participants

We will recruit patients from eleven Dutch medical centres (i.e. hospital and clinics), from both urban and rural areas throughout the country (please see Appendix 2a for a full lists of participating centres). By including patients from different areas throughout the country, we aim to include a sample that is generalizable to the Dutch population of working age knee arthroplasty patients.

Patients within the working age (18–67 years) with a paid job for at least eight hours a week, who are on the waiting list for a primary total- or unicompartmental knee arthroplasty (i.e. including patients who already received a previous primary total- or unicompartmental knee arthroplasty in the other knee and were fully recovered) and are willing to return to work will be eligible to participate in our study. Factors that might affect the postoperative recovery in terms of societal participation or factors that might influence the intervention serve as exclusion criteria. Examples are extreme comorbidity that could influence the postoperative course or another joint replacement during the study period. An overview of all the inclusion- and exclusion criteria is presented in Appendix 2b.

### Recruitment of participants

Orthopaedic surgeons will be informed about the study and have a card with inclusion- and exclusion criteria. In each centre, patients on the waiting list for a knee arthroplasty will have a pre-operative consult with their orthopaedic surgeon (usual care), in which the procedure of the operation will be discussed. During this consult, the orthopaedic surgeon will inform potentially eligible patients about the study and will ask patients if they are interested in participation and for approval to share their contact information with the Amsterdam UMC research team. Also, patients on the waiting list for surgery will be called by either the orthopaedic surgeon or a delegated colleague of the orthopaedic surgeon. If patients agree, the orthopaedic surgeon or delegate will provide patients with an information package containing a patient information letter and study information (appendix 3), informed consent form and return envelope. The orthopaedic surgeon or delegate will provide the research team with the patient's contact information. After the patient's minimum reflection period of three days, the research team will contact the patient by phone and again assess the patient’s willingness and eligibility. Eligible and willing patients are requested to sign and return the informed consent form to the research team. After receiving the patient’s signed informed consent, the patient is included in the study.

### Randomization and allocation

Included patients will complete a first (baseline) questionnaire approximately eight weeks before surgery. Thereafter, randomization will be performed at the patient level. Randomization will be performed in a 1:1 ratio using computer-based randomization lists in Excel, developed by an independent statistician of the Epidemiology and Biostatistics department of Amsterdam UMC. Randomization will be pre-stratified by medical centre (with or without eHealth as usual care), operation procedure (total- or unicompartmental knee arthroplasty) and recovery expectations regarding return to work [25], either positive: “I expect to be fully back at work earlier than the average knee arthroplasty patient” or negative: “I expect to be fully back at work later than the average knee arthroplasty patient”. Only for the randomization lists for medical centres that do not have eHealth as usual care, block randomization will be used. The allocation sequence is predetermined by the statistician and cannot be altered by the researchers. The randomization will follow the order of receiving the patient’s digital baseline questionnaire. Patients will be blinded to the randomization to either the control- or the intervention group.

After allocation, each patient will receive an e-mail containing instructions and a link and log-in details to either the ikHerstel app or to the eHealth from their own hospital or clinic (as explained below in the intervention paragraph). The research team will inform the medical team of the corresponding centre and, if a patient is allocated to the intervention group, the patient’s case-manager. If any patient refuses or wishes to withdraw from the study, either during the inclusion period or during the study period, they will continue to receive care as usual only.

### Intervention

#### Development

The development of the care program under study involved various steps, including a systematic review with meta-analysis [14], focus groups with total knee arthroplasty patients [26], and a Delphi study with orthopaedic surgeons, physiotherapists, occupational physicians and a physician assistant [27] to develop the algorithms for the ikHerstel app.

Our systematic review showed that integrated care programs consisting of one or a combination of: an active referral to a case-manager, a patient tailored rehabilitation program using goal setting and/or eHealth, showed small effects on work- and/or sports participation post-surgery [14]. Moreover, previous studies by our research group showed positive effects on societal participation of integrated care programs with similar intervention elements among other patient populations, such as low back pain patients [28, 29] and gynaecological or abdominal surgery patients [30–32]. For the gynaecological and abdominal surgery patients, an eHealth intervention using the ikHerstel app was developed to support and advise them during their post-operative course. This app was previously found to be effective for returning to daily life activities, where patients in the intervention group returned to daily (work) activities 5–13 days earlier than those in the usual care group [30–32]. Because of the promising effectiveness results of the ikHerstel app, we have further developed this care program according to the wishes and preferences of knee arthroplasty patients with focus groups and an algorithm that was developed in a Delphi study [26, 27]. In the latter study, with an expert panel including orthopaedic surgeons, physiotherapists, occupational physicians and an orthopaedic physician assistant, we have developed uniform and multidisciplinary recommendations for the resumption of daily life activities after knee arthroplasty [27]. The recommendations were implemented in our smartphone ikHerstel app. Moreover, an activity tracker was linked to the ikHerstel app to enhance resumption of physical activity [33–35]. Goal attainment scaling was added to the intervention, given the promising results on satisfaction with occupational and leisure time physical activities among younger knee arthroplasty patients [36, 37].

Based on the above, in the ACTIVE trial, patients will receive an intervention consisting of three components: 1) a personalized eHealth intervention (the ikHerstel app) including an activity tracker, consisting of a mobile phone app available for Android and Apple devices, 2) goal setting using goal attainment scaling to improve rehabilitation and 3) a referral to a case-manager to secure and oversee an adequate start of the pre- and postoperative care. As our target population is patients of relatively young (< 67 years) age, we expect that an app will make our intervention more approachable for them. The protocol timeline for the enrolment and intervention components is outlined in Table 1.

**Table 1:**
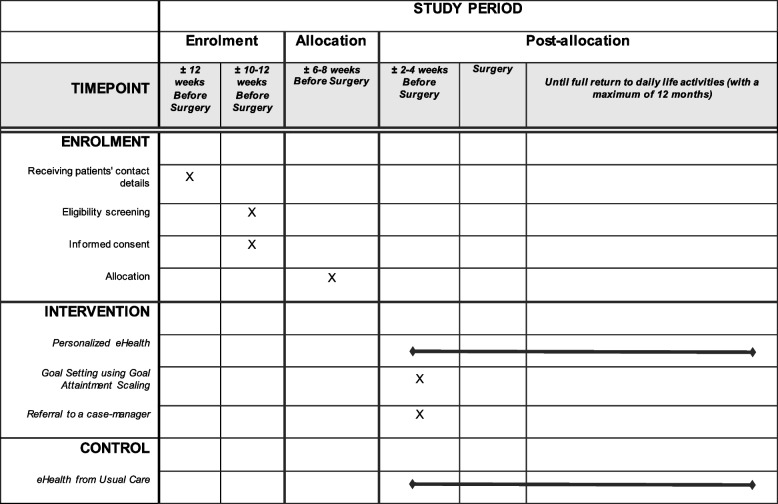
Protocol timeline

#### Component 1 – personalized eHealth

**Fig. 1:**
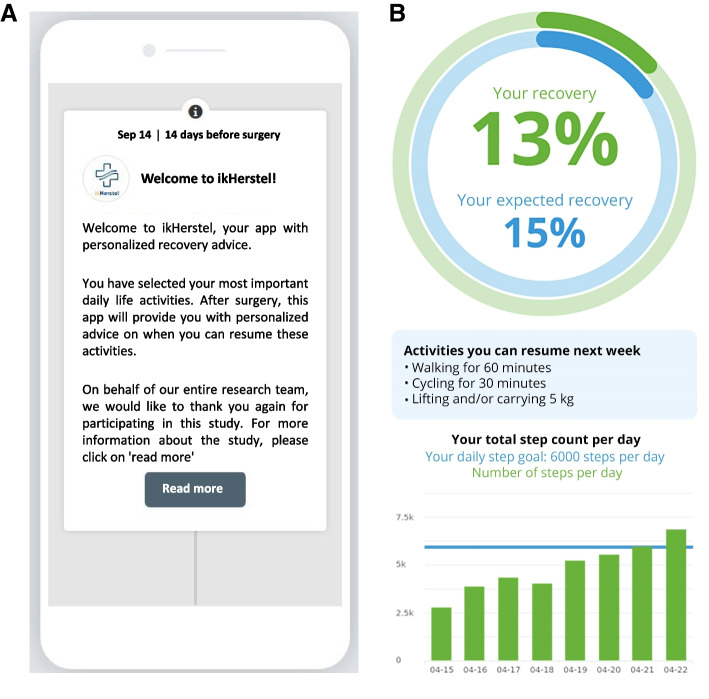
Screenshots of the ikHerstel app. Note, the original app is in Dutch. This screenshot provides an English translation of the original app. **A** Welcome message in the ikHerstel app **B** After completing the recovery monitor, a dashboard will provide patients with an overview of their recovery

The first component of our intervention is a patient tailored eHealth program, using the ikHerstel app (Fig. 1). Approximately two to four weeks prior to surgery, patients allocated to the intervention group will get access to the ikHerstel app including the activity tracker. The case-manager (see component 3) will talk patients through the ikHerstel instructions. Prior to their surgery, personal data concerning the patient’s surgical procedure and the day of surgery will already be filled in by the researcher or research assistant. Hence, when patients log in to the app, it will already provide them with personalized information. For instance, patients will receive pop-up messages on their mobile phone with important notifications of the app, including certain advices or guidance. Patients can use the app according to their own preferences, e.g. there is no minimal time that they need to spend on the app.

Pre- and postoperatively, the ikHerstel app aims to guide patients through their recovery period by providing guidance and feedback on their recovery to facilitate return to their personal daily life activities. The app consists of the following main elements:

- Preoperative information, with the goal to prepare the patient for their upcoming surgery and to improve recovery expectations. Previous research has concluded that the length of recovery might be reduced by having clear expectations about the recovery period and resumption of daily activities [15, 32]. The app will provide: 1) preoperative information such as a packing list and recommendations for preparations at home, 2) information about the admission day and the surgical procedure, and 3) general and practical information about the recovery period, such as commonly experienced post-operative complaints and frequently asked questions. All information will be provided in text and will be supported by short video’s (Fig.1A).
- Self-management and empowerment of patients by personalized perioperative recovery recommendations, developed by our research team in a Delphi study [27]. Preoperatively, patients have the opportunity to select daily life activities that are important in their daily life from a list of 26 activities. Before the surgery, patients will receive their recommendations in a personalized recovery plan with advice on when and how to resume their selected activities. Patients will discuss their recovery plan with their case-manager. Postoperatively, patients will receive these personalized recovery recommendations in the app with notifications, videos and text messages.
- Monitoring personal patient recovery after the surgery. Once a week, patients will complete a recovery monitor in the app. The recovery monitor is a short questionnaire in which patients answer if they have resumed (one or more of) their daily life activities. Abovementioned recovery recommendations will be personalized based on the stage of the recovery the patient is in. The underlying algorithm makes it possible to alter the patients recovery recommendations if needed throughout the recovery by distinguishing three rates of recovery: fast, average and slow. Preoperatively, patients will be categorized into one of those three recovery rates based on their recovery expectations regarding return to work (i.e., after how many days, weeks or months do patients expect to be fully back at work). Each recovery rate has its own corresponding recovery recommendations that were developed in the aforementioned Delphi study [27]. Patients who are categorized in the recovery rate ‘fast’ have more progressive recommendations as compared to patients who are categorized in the recovery rate ‘average’ and ‘slow’. Postoperatively, based on a patient's answers in the recovery monitor, a patient can shift to a faster or slower group. If patients show a delayed recovery (e.g. they can only resume < 33% of the activities that they should be able to), the algorithm will switch patients to a slower group (if not yet in the slowest group) and the patient's recovery recommendations will be altered. If patients can resume 33% to 66% of the activities they should, they remain in their group. If patients can resume 67% or more of their given recommendations, patients will switch to a faster group (if not yet in the fastest group). This allows us to provide personalized and adaptive recovery recommendations. The patient is not informed about this change (i.e. shifting to a slower or faster group), but will receive altered recommendations. The recommendations of the patient's personal recovery plan will be updated automatically every four weeks in the app, and if recommendations are adapted, a new recovery plan will be send to the patients by email.
- An accelerometer based activity tracker (Xiaomi Mi Band 4/6 or, if compatible, patients’ own activity tracker) will be linked to the app. Previous research has concluded that an accelerometer is a feasible instrument for measuring postoperative recovery of physical activity [33–35, 38]. The accelerometer can be worn on the wrist and will measure daily step count. Patients will be requested to wear an activity tracker from two weeks before surgery until six months after surgery to monitor their physical activity recovery and intensity levels. Four weeks after surgery, a daily step goal will be implemented in the ikHerstel app. Based on previous studies by van der Walt et al. [39], Hylkema et al. [34], Hoorntje et al. [35] and Lebleu et al. [40], we developed the following post-surgical step goals: 3000 + steps after four weeks, 4000 + steps after six weeks, 5000 + steps after eight weeks and 6000 + steps after ten weeks. Every day, patients can see their progress regarding their daily step goal and they will receive an overview of their steps once a week.
- Feedback on their recovery, among others by short videos in which either an orthopaedic surgeon, a physiotherapist or an occupational physician provides them with guidance on how and when best to resume activities of daily life. A dashboard will display the recovery percentage appearing after completing the weekly recovery monitor (Fig. 1B).

#### Component 2 – goal setting using goal attainment scaling

The second component involves patient-specific participation goal attainment scaling (GAS). GAS is a valid and reliable tool for facilitating patient-centred rehabilitation care, and has been used for setting patient-centred goals during postoperative rehabilitation [37]. In this way, improvement towards these individual goals and activities can be easily assessed, while potential problems during the rehabilitation can be identified.

During their appointment with their case-manager (component 3), patients will define a specific GAS goal for their post-surgical rehabilitation. This goal has to be related to their work-activities and thus return to work, such as walking stairs at work or lifting boxes during the workday, given that defining such goals have shown to be effective for patient satisfaction regarding their recovery [36]. The case-manager will collaborate with the researchers to validate the patient’s GAS goal. Patients are stimulated to discuss their GAS goal with their physiotherapist in order to achieve their GAS goal and are also stimulated to discuss their goal with their occupational physician, family and friends.

#### Component 3 – active referral to a case-manager

The last component is an active referral to a case-manager, e.g. physiotherapist, nurse practitioner, or a member of the research team. Patients will meet their case-manager approximately two to four weeks prior to surgery. The case-manager will secure and oversee an adequate start of the pre- and postoperative care by:

- Providing instructions and support regarding component 1 and 2 of the intervention, i.e. installation and instructions for the ikHerstel app and activity tracker and determining a GAS goal.
- Exploring and discussing the patients’ recovery expectations based on the patients’ own activity preferences and recovery plan of the ikHerstel app;
- Informing patients about what to expect from their recovery using their personal recovery plan.

#### Usual care

Patients allocated to the control group will receive pre- and postoperative care as usual according to protocols from their hospital or clinic. All types of care as usual are included. According to the guidelines of the Dutch Orthopaedic Association (NOV), usual care for patients eligible for knee arthroplasty is usually limited to surgical placement of an implant, supplemented by 1) a pre-surgery evaluation by the surgeon and/or physician assistant, 2) pharmaceutical treatment, such as antibiotics or pain relieve medication, and 3) post-surgery clinical check-ups, mainly targeted at wound healing and implant position. This usual care is covered in the Dutch healthcare insurance. Depending on the patient and his/her healthcare insurance, this can be supplemented by physical rehabilitation. Moreover, participants will receive access to eHealth from care as usual. This can consist of an app from the hospital or clinic (such as the Patient Journey App) or a website, depending on the patient’s hospital or clinic. The usual care apps mainly consists of information such as appointment dates, contact information, wound healing information and sometimes rehabilitation exercises. The website shows similar information. For the sake of clarity, the usual care apps and website do not focus on postoperative return to daily activities including work and sports.

### Blinding

Patients will be blinded as both groups receive a form of eHealth, however with a different dose delivered. It will be impossible, however, to blind the researchers, health care providers and case-managers during the data collection, as they will be in contact with patients allocated to the intervention group. After data collection, the data will be pseudonymized and data will be blinded for the researchers during the analyses.

### Sample size calculation

We based our sample size calculation on the effect size from a comparable intervention and patient sample [29], and α = 0.05; Power = 80% and an Intraclass Correlation Coefficient (depicting correlation within surgeons) of 0.010. Using a conservative estimation of the effect size, we expected a hazard ratio (HR) of 1.7 and 50% of the patients to return to activities of daily life by the end of the follow-up period in the group who receive our intervention compared to care as usual. Considering this HR, using a 2-sided log-rank test and incorporating clustering of our study (with an estimated 10 patients per surgeon), we need to observe 220 patients. To take 20% dropout into account, we need to recruit 276 patients (i.e. 138 per arm). Agreements were made with all participating centres in which they committed to recruit a certain number of patients, varying between 20 and 80 patients per participating centre.

### Outcomes

The primary outcome measure of this study will be quality of life, measured with the personalized and reliable, validated and responsive Patient-Reported Outcomes Measurement Information System – Physical Functioning (PROMIS-PF) item bank [20, 41, 42]. A list of 29 activities from the item bank will be presented to the patients at the baseline measurement, from which patients will be asked to select eight activities which are most relevant for them in daily life. By doing this, patients will design their own quality of life short form.

Each activity consists of five functioning levels. Seventeen of the 29 items are scored as: without any difficulty, with a little difficulty, with some difficulty, with much difficulty and unable to do. Twelve items are scored as: not at all, very little, somewhat, quite a lot, and cannot do. At baseline, patients will report their current functioning level. The personalized short form will also be presented to the patients during all follow-up measurements. Post-surgery, successful ‘return to an activity’ is defined by a return to an activity with at least one functioning level higher as compared to the baseline (pre-surgery) functioning level, for instance from ‘with much difficulty’ to ‘with some difficulty’. If patients reach this level, they will be asked at what day they reached this level. Our primary outcome will be defined as the time since surgery to resume six out of the selected eight activities of their own quality of life short form. The secondary outcomes that will be assessed are shown in Table 2.

**Table 2:** Secondary outcomes

Return to work	The time (i.e. days, months) between the surgery and the first day back at work, both partly and fulltime
Knee Functioning, measured with The Knee Injury and Osteoarthritis Outcome Score (KOOS) [43]	The KOOS consists of 42 items that assesses five outcomes: pain (9), symptoms (7), activities of daily living (17), sport and recreation function (5), and knee-related quality of life (4) on a 5-point Likert scale (0, no problems – 5, extreme problems). Scores are transformed to a scale of 0 – 100, with 0 representing extreme knee problems and 100 representing no knee problems
Pain intensity, measured with the von Korff questionnaire visual analog scale (VAS) [44]	The pain VAS is a unidimensional measure of pain intensity and is widely used in postsurgical patient populations. This questionnaire measures the severity of pain on a scale of 0 (no pain) to 10 (extreme pain) and the associated impediment with daily life activities
Health-related quality of life, measured with the 5-level EuroQol-5d (EQ-5D-5L) [45]	The EQ-5D-5L contains five questions that represent the following health dimensions: mobility, self-care, usual activities, pain/discomfort and anxiety/depression. For each dimension, patients have to indicate the severity of their health complaints. There are five severity levels: normal/no problems, slight problems, moderate problems, severe problems, and extreme problems, all scored from 1 (no problems) to 5 (extreme problems). Hence, answers to these questions will represent a patient’s EQ-5D-5L health state, ranging from 11,111 (no problems on all dimensions) to 55,555 (extreme problems on all dimensions)
Physical difficulty experienced at work, measured with the Work, Osteoarthritis or joint-Replacement Questionnaire (WORQ) [46]	The WORQ is a valid and reliable questionnaire that can be used to assess the impact of the knee replacement. It contains 13 questions, each representing a physical activity (e.g. kneeling, crouching or standing). Patients have to grade how difficult this activity is to perform on a five-point scale: none, mild, moderate, sever of extreme – corresponding with scores of 4 to 0, respectively. The sum of scores is then converted into a score of 0 (worst score) to 100 (best score)
Fatigue, measured with the Multidimensional Fatique Inventory (MFI-20) [47]	The MFI-20 is a valid and reliable self-report instrument that has been designed to measure fatigue in several dimensions: general fatigue, physical fatigue, reduced activity, reduced motivation and mental fatigue. Each dimension has five questions rated on a five-point Likert scale. Scores on each subscale range from 4 to 20, with higher scores indicating greater fatigue. The sum of the subscale scores represents the total fatigue score (range 20 – 100), with a higher score indicating a higher level of fatigue

### Patient characteristics

In addition to the aforementioned primary and secondary outcomes, several demographic factors and patient characteristics will be assessed at baseline: age, sex, body mass index, socio-economic position, total- or unicompartmental knee arthroplasty, work status (employed or self-employed), knee demanding work, work absenteeism prior to surgery, patient expectations regarding return to work after surgery, work-related knee complaints (i.e. knee complaints during knee demanding activities at work) and self-reported work-relatedness of symptoms. These factors can be adjusted for as potential confounding variables in the analysis.

### Process measures

A process evaluation of the intervention will be conducted according to method described by Linnan and Steckler [48], to identify effective key components of the intervention and/or components that should be improved for further implementation. Data for the process evaluation will be collected using information from the study procedure database, a log of the ikHerstel app and an online patient satisfaction questionnaire that will be administered at 6 weeks and 6 months follow-up. The process measures that will be assessed are shown in Table 3.

**Table 3:** Process measures

	**ikHerstel eHealth**	**Goal attainment scaling**	**Case-manager**
**Reach** *The degree of participation*	Eligible participants that were 1) interested in participation and 2) were included and randomized to the intervention or usual care control group^a^ as a percentage of the target population. The estimation of the target population is based on the number of eligible patients that had a consult with their orthopaedic surgeon in the participating centres during the inclusion period		
**Dose delivered** *The proportion of the intervention that was delivered to the patients*	The percentage of patients in the intervention group who received log-in details for the ikHerstel app^a^	The percentage of patients in the intervention group that were invited to make a goal attainment scaling goal before surgery^a^	The percentage of patients in the intervention group who got referred to a case-manager^a^
**Dose received** *The “exposure” and usage of the intervention components*	The percentage of patients in the intervention group that -made a personal recovery plan^b^ -logged in to the ikHerstel app^b^ -completed a recovery monitor^b^ -connected their activity tracker to the app^b^	The percentage of patients in the intervention group that made a work-related goal attainment scaling goal before surgery^a,b^	The percentage of patients in the intervention group that had an appointment with their case-manager before surgery^a,b^
**Patient attitudes** *Patient satisfaction and barriers for the usage of the intervention*	-The mean score that patients rated the ikHerstel app on a 1 – 10 scale^c,d^ -Tabulation of reasons for using and/or not using the ikHerstel app^c,d^	The mean score that patients rated their satisfaction about goal attainment scaling on a scale of 1 – 10^c^	The mean score that patients rated their satisfaction about the appointment with their case-manager on a scale of 1 – 10^c^

^a^study procedure database

^b^log of the ikHerstel app

^c^online questionnaire at 6 weeks follow-up

^d^online questionnaire at 6 months of follow-up

### Costs

An economic evaluation will be executed according to the guidelines of the Dutch National Health Care Institute (Zorginstituut Nederland, 2016). The economic evaluation will be performed from a healthcare and societal perspective. For the healthcare perspective, only costs accruing to the formal Dutch healthcare sector will be considered. For the societal perspective, we will consider costs of the intervention, other healthcare services, occupational healthcare services, informal care, unpaid productivity, absenteeism, and presenteeism.

All relevant costs will be measured using online cost-questionnaires with 3-month recall periods. The cost-questionnaire was developed by our department and tailored to the population and intervention under study. Intervention costs include all costs related to the development, implementation, and execution of the intervention (e.g. ikHerstel, GAS and case-manager) and will be estimated using a micro-costing approach. That is, the cost estimate will be based on actual resources depleted, which will be assessed in detail using prospective data collection, and will be valued in accordance with the Dutch manual for costing studies in health care [49, 50]. Other healthcare costs will consist of costs of primary care (e.g. family physician), secondary care (e.g. hospital stay and visits), and medication (both prescribed and over the counter). The cost of occupational healthcare services will consist of costs of visits to the occupational physician or occupational (physio)therapist. If available, other and occupational health care costs will be valued using Dutch standard costs [50]. If unavailable, prizes of professional health care organizations will be used. Medication use will be valued using prices derived from http://www.medicijnkosten.nl. Informal care will be measured by asking patients to report the number of hours per week they received help from family and friends. Unpaid productivity losses will be measured by asking patients to report the number of hours per week that they were unable to perform unpaid activities (e.g. voluntary work). Unpaid productivity losses and informal care will be valued using a recommended Dutch shadow price [50]. Absenteeism (e.g. total number of sick leave days, measured using a slightly adapted version of the iPCQ [51]) and presenteeism (e.g. lower productivity as compared to normal while at work, measured with the WHO-HPQ [52]) will be valued using sex-specific price weights [50]. Using consumer price indices, all costs will be converted to the same reference year. Because the follow-up period of this study is 12 months, it is not necessary to discount costs and effects.

### Measurements

Digital questionnaires will be administered with the Survalyzer online tool (survalyzer.com), which can be accessed through a link provided via e-mail, to assess all primary and secondary outcome parameters and additional variables at baseline, and 3, 6, 9, and 12 months after surgery. Measurements will be taken from both patients in the intervention and usual care group, regardless of whether they actually followed the intervention (when in the intervention group). The primary outcome parameter and return to work will also be measured every month during the first 6 months. Six weeks after surgery, we will also assess knee functioning (KOOS), pain (VAS), quality of life (EQ-5D-5L) and patient satisfaction. Table 4 shows an overview of our outcome measures, measurement instruments and measurement moments. All relevant costs will be measured using online cost-questionnaires administered at baseline, 3, 6, 9, and 12 months post-surgery.

**Table 4:** Outcome measures, measurement instruments and measurement moments

	**STUDY PERIOD**									
**TIMEPOINT**	***t***_***0***_	***t***_***1***_	***t***_***2***_	***t***_***3***_	***t***_***4***_	***t***_***5***_	***t***_***6***_	***t***_***7***_	***t***_***8***_	***t***_***9***_
*** Primary outcome***										
***Quality of life - Duration of return to daily life activities (PROMIS)***	X	X	X	X	X	X	X	X	X	X
*** Secondary Outcomes***										
***Return to Work***		X	X	X	X	X	X	X	X	X
***Physical difficulty experienced at work (WORQ)***	X	X	X	X	X	X	X	X	X	X
***Disease specific functional status (KOOS)***	X		X		X			X	X	X
***Pain Intensity (VAS)***	X		X		X			X	X	X
***Quality of Life (EQ-5D-5L)***	X		X		X			X	X	X
***Energy/Fatique (MFI-20)***	X				X			X	X	X
*** Patient characteristics***										
***Demographics and Patient Characteristics***	X									
*** Process***										
***Patient Satisfaction***			X					X		

T_0_ = baseline, ± 6–8 weeks before surgery; T_1_ = 4 weeks after surgery; T_2_ = 6 weeks after surgery; T_3_ = 2 months after surgery; T_4_ = 3 months after surgery; T_5_ = 4 months after surgery; T_6_ = 5 months after surgery; T_7_ = 6 months after surgery; T_8_ = 9 months after surgery; T_9_ = 12 months after surgery

Patients will have two weeks to complete the questionnaire. After approximately one week, a reminder will be sent by e-mail. If after two weeks the questionnaire is not yet completed, an attempt will be made to contact the patient by phone to encourage them to still complete the questionnaire.

### Statistical analyses

Analyses will be performed in SPSS (effect evaluation) and STATA or R (economic evaluation). All analyses will be performed according to the intention to treat principle. If required, missing cost and effect data will be imputed using multiple imputation according to the MICE algorithm developed by van Buuren and colleagues [53]. To account for the possible clustering of data (with data clustered by surgeon), analysis will be performed using linear multilevel analysis. Accounting for the possible clustering of data is important, as many (economic) evaluations fail to do so, whereas ignoring the possible clustering of data might lead to inaccurate levels of uncertainty and sometimes even biased point estimates [54]. Sensitivity analyses will be performed to test the robustness of the study results. Statistical analyses will be performed according to intention-to-treat principle, which will be compared to per-protocol analyses. Patients will be included in the per-protocol analyses when they used the intervention as intended, which will be defined as having an appointment with their case-manager, defining a GAS goal and generating a personal recovery plan in the ikHerstel app. This will be measured with the study procedure database and a log of the ikHerstel app. Characteristics of the patients measured at baseline will be evaluated and summarized using descriptive statistics.

### Effect analysis

The effect of the intervention on the primary outcome parameter quality of life (i.e. duration until return to activities of daily life, PROMIS-PF) and the secondary outcome time until return to work will be assessed using a multi-level longitudinal Cox proportional hazard model, estimating hazard ratios (HR) and their 95% confidence intervals (95%CI). Multi-level longitudinal linear regression will be used to assess the effect of the intervention regarding all continuous secondary outcomes (e.g. knee functioning, pain intensity, health-related quality of life, physical difficulty experienced at work, and fatigue) estimating betas and their 95% CI. In all models, repeated measurements will be taken into account. We will test the effect of intervention, time and intervention*time. Based on previous literature, several variables will be considered as potential confounders or effect modifying variables, including body mass index, knee demanding work, socio-economic position, work absenteeism prior to surgery, self-reported work-relatedness of symptoms, first primary or second primary knee arthroplasty and the variables used for stratification (unicompartmental- or total knee arthroplasty, participating centres with or without eHealth as usual care, and recovery expectations). Potential confounders will be tested using a forward stepwise selection procedure. If adding the confounder to the model leads to an effect size difference of 10% or more, the variable will be considered as confounder and will be included in the final model. Effect modification will be tested using interaction terms with the intervention (i.e. being/not being in the intervention group). In case of statistically significant interaction terms, analyses will be stratified by the subgroup.

Lastly, we will repeat our analysis to investigate the effect of the intervention on quality of life and return to work within subgroups of patients that, in addition to our intervention, did or did not receive eHealth as usual care from their own hospital or clinic.

### Economic evaluation

The economic evaluation will be performed for the primary outcome (quality of life) and quality-adjusted life-years (QALYs). For estimating quality adjusted life years (QALYs), the patients’ EQ-5D-5L health states will be converted into utility scores using the Dutch tariff [55]. Subsequently, QALYs will be estimated using linear interpolation between the measurement points.

To account for the highly skewed nature of cost data, the Bias Corrected and Accelerated Bootstrap method (5000 replications) will be used to estimate 95% CIs around the differences in costs. Incremental Cost-Effectiveness Ratios (ICERs) will be calculated by dividing the differences in costs by the differences in the primary outcome quality of life and QALYs. For a graphical illustration of the joint uncertainty surrounding costs and effects, bootstrapped cost-effect pairs will be plotted on cost-effectiveness planes. Cost-Effectiveness Acceptability Curves will also be presented, indicating the intervention’s probability of being cost-effective compared with the control for a range of willingness to pay values. Willingness-to-pay values represent the maximum amounts of money that the decision-makers (i.e. relevant stakeholders) are willing to pay per extra unit of effect.

### Harms

All (serious) adverse events (i.e. undesirable experiences occurring to a subject during the study) related to the intervention (e.g. complications during or after resuming daily activities according to the recovery plan), reported spontaneously by the patient or observed by the investigator (i.e. research team) or the orthopaedic surgeon, will be recorded in our study database, reported in our article on (cost-)effectiveness and, if necessary, reported to the Medical Ethical Committee of Amsterdam UMC, location VUmc. If unblinding of the allocation is necessary, we will do so.

### Project management

We aim to report findings from this study in peer-reviewed scientific journals. The current author team will be leading authors on these articles, possibly supplemented by representatives from the participating centres, and only if Vancouver and Amsterdam UMC publishing guidelines are met. Pseudonymized participant data will be stored on secured Amsterdam UMC servers. Files will only be accessible for the researchers and keys to identify participants will be saved in a separate location, locked with a password. Data will be saved for 15 years, as signed for in the informed consent form. Regular monitoring of the data and procedures will be done in accordance to Dutch legislation for medical research by an independent monitor and in accordance to the monitoring plan developed a-priori by the researchers. After study completion, we strive to make data available for future research through a repository.

## Results

The inclusion process started in October 2020 and will probably last until March 2023 given the delay in inclusion due to COVID-19. With a follow-up period of one year, data collection is expected to be finished in 2024, after which analyses and reporting will commence. The study is aimed to be finalized at the end of 2024.

## Discussion

In this multicentre RCT we will evaluate the (cost-)effectiveness of a personalized multidisciplinary eHealth program supporting societal participation in knee arthroplasty patients versus care as usual. Our hypothesis is that the intervention will be more effective regarding the primary outcome quality of life, measured by the duration until return to activities of daily life, and cost-effective compared with usual care.

### Strengths and limitations

This integrated care program is based on extensive preliminary research, including our systematic review on transmural care, focus groups assessing the experiences and wishes of knee arthroplasty patients, earlier research on the ikHerstel app in other patient populations (i.e. gynaecological and abdominal surgery patients), our Delphi study [27] in which we used expert input to develop an algorithm to tailor the eHealth app to knee arthroplasty patients and our studies showing the effectiveness of GAS among this patient group. In these studies, our care program has been critically evaluated and adapted with the help of the involved stakeholders. Another major strength of this integrated care program is the innovative tailored ikHerstel app with the possibility to alter the recommendations for each unique patient, providing more encouragement and relevance to the patient. Furthermore, recovery recommendations will be provided during the first twelve months after surgery, the period during which work participation has shown to occur [13]. And, the nature of our eHealth intervention allows for patients to receive remote care, which has become more relevant in the past years with limitations to travel and visit health care facilities due to the COVID pandemic.

Using PROMIS short forms as primary outcome measurement instrument, we use a form of tailored testing. PROMIS has proven effective as a reliable and valid measurement to assess participation using adaptive testing, in which the selection of items presented to the patient are based on the patient’s own response [42]. This is important to be able to compare the outcomes of various patients with different needs. Additionally, the access to eHealth form care as usual for the control group enables us to blind the patients, leaving them unknown to which group they are allocated. Last, due to the wide diversity of participating hospitals and clinics (academic, teaching, nonteaching, rural and urban), the generalizability of this study will probably be high and will provide a heterogeneous sample of included patients.

A few limitations of this study also need to be addressed. First, it is not possible to blind health care professionals in this study, leaving our study with a risk of contamination and bias on this aspect. Orthopaedic surgeons might hypothetically treat patients differently and provide either more or less attention to return to daily life activities. However, previous research has shown that the actual bias due to this is rather limited, and a meta-epidemiological study of Moustgaard et al. [56] showed no average difference in estimated treatment effects in trials with and without blinding of health care professionals. Moreover, the health care professionals will not be involved in data collection, which will likely attenuate the risk of bias in our results. Second, the heterogeneity of our participating centres could also be seen as a disadvantage, as the provided usual care for the control group might vary between centres. We will account for this bias by stratifying the randomization by participating medical centres with and without eHealth. Moreover, in our analyses we will, by using multi-level modelling, statistically adjust for the clustering within surgeons. Furthermore, as this study will only include (self-) employed patients, it might be argued that unemployed patients cannot benefit from our care program. However, it is important to note that this intervention not only aims at an accelerated return to work but also aims to improve return to daily life activities, making this intervention possibly usable for patients of all ages that receive a knee arthroplasty in a future study. Lastly, this intervention was tailored for Dutch orthopaedic patients (using the Dutch –occupational- healthcare system as context), so generalizability to other countries needs to be done with due caution.

## Conclusion

With the rising obesity epidemic and the aging population, resulting in a rapidly increasing knee arthroplasty prevalence worldwide [7], it is essential to deliver (cost-)effective care that results in enhanced societal participation of these patients compared to care as usual.

With an accelerated return to daily activities, including work and sports after knee arthroplasty, patients can expect a more timely and better quality of life. As a result, this will potentially benefit employers and society as a whole by reducing costs due to productivity loss and sick leave. The results from this study will provide health care providers and policy-makers with guidance to improve arthroplasty patients’ care and future implementation of this integrated care program in Dutch orthopaedic practices and hospitals. Finally, the results of this study will also provide more insight in determining whether perioperative care using eHealth and GAS can be used to provide additional effective care or may even substitute care as usual against lower costs.

## Supplementary Information

**Additional file 1:** **Appendix 1.** SPIRIT checklist.

**Additional file 2:** **Appendix 2.** An overview of the participating hospitals and inclusion- and exclusion criteria.

**Additional file 3:** **Appendix 3.** Participant information for participation in medical scientific research.

## Data Availability

Not applicable. No data are presented in this article.
