# Supplementary material for: (Cost-)effectiveness of a personalized multidisciplinary eHealth intervention for knee arthroplasty patients to enhance return to activities of daily life, work and sports – rationale and protocol of the multicentre ACTIVE randomized controlled trial
Source: BMC Musculoskelet Disord. 2023 Mar 4;24:162. doi: 10.1186/s12891-023-06236-w (PMC9984288; doi:10.1186/s12891-023-06236-w)
Supplement: Supplementary file 2 — Additional file 2: Appendix 2. An overview of the participating hospitals and inclusion- and exclusion criteria. [file 12891_2023_6236_MOESM2_ESM.docx]

**Appendix 2: An overview of the participating hospitals and inclusion- and exclusion criteria**

| Table 2a. Participating Centres |
| --- |
| Amphia, Breda |
| Anna Ziekenhuis, Geldrop |
| OLVG, Amsterdam |
| MUMC, Maastricht |
| ViaSana, Mill |
| Flevoziekenhuis, Almere |
| Máxima Medisch Centrum, Eindhoven |
| Alrijne, Leiden |
| Catharina Ziekenhuis, Eindhoven |
| Gelderse Vallei, Ede |
| Bergman Clinics, Capelle aan de IJssel |

| Table 2b. Eligibility criteria |  |
| --- | --- |
| Inclusion criteria | Exclusion criteria |
| Patients scheduled for primary total or unicompartmental knee arthroplasty | Pregnancy |
| 18-67 years of age | Combination of several surgical procedures |
| Paid job ≥ 8 hours/week, either employed or self-employed | Knee replacement due to another cause than severe arthritis |
| Intention to return to work after the surgery | Other neuromuscular disease influencing the lower extremities |
|  | Other planned joint replacement during study period |
|  | Serious psychiatric disorder(s) |
|  | Severe comorbidity that could influence post-operative recovery |
|  | Unable to understand informed consent and patient information |
|  | Insufficient understanding of Dutch language |
