# Supplementary material for: (Cost-)effectiveness of a personalized multidisciplinary eHealth intervention for knee arthroplasty patients to enhance return to activities of daily life, work and sports – rationale and protocol of the multicentre ACTIVE randomized controlled trial
Source: BMC Musculoskelet Disord. 2023 Mar 4;24:162. doi: 10.1186/s12891-023-06236-w (PMC9984288; doi:10.1186/s12891-023-06236-w)
Supplement: Supplementary file 3 — Additional file 3: Appendix 3. Participant information for participation in medical scientific research. [file 12891_2023_6236_MOESM3_ESM.docx]

**Appendix 3: Participant information for participation in medical scientific research**

**Recovery program after knee arthroplasty**

*Official title: The (cost)effectiveness of a transmural intervention program for knee arthroplasty patients in the working population*

**Introduction**

Dear Sir / Madam,

Soon you will receive a total- or unicompartmental knee arthroplasty. In order to improve the care for similar patients in the future, we ask you to participate in a medical-scientific study.

Participation is voluntary. Your written consent is required to participate. Before you decide whether you want to participate in this study, you will receive an explanation of what the study entails. You have at least three days to consider whether you want to participate. Then we will call you. Please read this information carefully and ask the researcher for clarification if you have any questions. You can also ask the independent expert named at the end of this letter for additional information. You can also talk about it with your partner, friends or family. Further information about participating in medical research can be found at www.rijksoverheid.nl/mensenonderzoek.

**1. General Information**

This study was set up by the Amsterdam UMC, location VUmc, and is being done by orthopaedic surgeons in 11 hospitals and/or clinics in the Netherlands. About 300 patients will participate in the study. The medical ethics review committee of VUmc has approved this study, and local permission has also been received from every hospital and clinic.

**2. Purpose of the research**

Two different eHealth care programs have been developed for this research. eHealth uses digital information technology, such as a website or app, to support healthcare. The aim of this study is to compare these two developed forms of support for the recovery of knee arthroplasty. For both care programs we study and compare the degree of recovery, return to your normal activities including work, quality of life and the costs to society. The aim of this study is to investigate which care program gives the best results.

**3. Background of the research**

Good support after surgery is an important condition for a good recovery. At present, there is no clear guidance for the recovery phase after knee arthroplasty.

Advice that patients receive about resuming their daily (work) activities can also differ. It is also often not clear when and who patients should contact after discharge from the hospital or clinic. The recovery may therefore be less successful than desired. It is estimated that approximately 57,900 knee arthroplasties will be performed in the Netherlands in 2030, so it is important to improve the guidance for these patients. In this study, two different care programs are offered that aim to promote recovery after knee arthroplasty and the return to daily (work) activities. Both care programs use eHealth, where you receive digital support in addition to the usual care. You can think of a website or app.

**4. What participation means**

This study has a duration of 12 months. We ask you to complete an online questionnaire about your recovery and return to work 10 times during these 12 months. These are 4 short questionnaires that last about 5 minutes, and 6 questionnaires that last an average of 30 minutes.

*Fitness*

First we determine whether you can participate. If you wish to participate, you will receive a short questionnaire. In this questionnaire, the researcher asks about your reason for the operation, work situation, and asks for some medical information that is relevant to this study, such as having another medical condition that may hinder your recovery. It is also important for this study that you perform paid work for at least 8 hours a week. You may therefore not be able to participate in the study, because otherwise the patients in the two groups differ too much. To be clear: you do not need to perform any physical tests.

*Care program*

You will receive one of the two eHealth care programs until you have fully resumed your daily activities, or until the end of the study period – which is 1 year after your surgery. Half of the patients will participate in both care programs. Based on a 50:50 chance it is determined which of the two care programs you receive. This draw results in two equal groups, whereby the results can be compared. The degree and form of supervision and care program you will receive will only be decided after you have decided to participate in the study. You will then receive login details for one of the two care programs. You do not know which group you are in. We want to prevent this from influencing the outcome of the research. If it is important for your health to know which care program you receive or received, we will let you know.

*Visits and measurements*

You may need to come to the hospital or clinic one extra time before your operation for a meeting with a case manager. This visit will take approximately 1 hour. During this appointment you will receive additional information about your eHealth care program, discuss your goals for resuming daily life activities after the surgery and you can ask questions. During this study, we would also like to gain insight into the physical recovery of patients after knee arthroplasty. We can measure this with an activity tracker. You may receive an activity tracker from us, and we will ask you to wear this activity tracker for a number of months, namely: from 2 weeks before the operation to 6 months after the operation. During this period we ask you to wear the activity tracker at least 5 days a week. This activity tracker is small and you can wear it on your wrist, so it will not bother you. It also provides you with insight into the recovery of your own physical activity.

As mentioned, both care programs require you to complete 10 questionnaires during 12 months – 4 short questionnaires of 5 minutes, and 6 questionnaires of 30 minutes. Depending on your preference, you will receive it on paper or digitally. The questions will be about your quality of life, resumption of daily activities, your pain complaints and your work situation. You will also be asked if and when you have achieved your personal goal, so that we can evaluate your recovery process.

*Different from usual care*

Normally you will see your orthopaedic surgeon or a colleague once or twice before the surgery and have a check-up appointment after your surgery. This is the usual care that your hospital or clinic offers to all patients. You will also have these visits during this study. The possible one-time visit prior to the surgery, the eHealth care program, and the possible activity tracker are additional. What is also different from the usual care is that we ask you to complete a questionnaire about your recovery 10 times over a period of 12 months.

**5. What is expected of you**

In order for the research to run smoothly, it is important that you agree to the following agreements.

The agreements are that you:

• Make the best possible use of the care program. This means that you log in to the eHealth care program to read your recovery advice daily to weekly. This will take about 15 minutes a day at first, but the time needed will decrease during the recovery period;

• Not also participate in another medical-scientific research that could influence the results of this research. If necessary, talk to your hospital or clinic or one of the researchers.

It is important that you at least contact the researcher:

• if you are admitted or treated in a hospital or clinic;

• if complications arise with your knee;

• if you suddenly develop health problems;

• if you no longer wish to participate in the study;

• if your contact details change.

*Pregnancy – applicable to women only*

Women who are pregnant during the study cannot participate. This is because a pregnancy can affect the return to work in particular. Breastfeeding women can participate in this study.

Do you become pregnant during the study? Please inform the researcher immediately. This research cannot have any consequences for an unborn child, but it can influence the outcome of our research - it will therefore be decided in consultation whether you can continue to participate in this study.

**6. Potential Adverse Effects/Discomforts**

The care programs to be examined will give you advice on your recovery. As far as is known, there are no side effects or adverse effects of this.

**7. Possible advantages and disadvantages**

It is important that you carefully weigh the possible advantages and disadvantages before you decide to participate in this study.

If you participate in this study, it means that you will receive a care program that in any case contains all elements of usual care, and is also aimed at returning to daily (work) activities. A possible advantage for you is that you receive extra guidance regarding your recovery, where the degree of guidance depends on the care program. You also contribute to more knowledge about recovery after knee arthroplasty, which will benefit future knee arthroplasty patients.

A disadvantage of participating in the study may be that it will take you extra time to complete the questionnaires. During the 12-month study period, you will spend a total of approximately 10 hours participating in this study. In addition, it may mean that you have an extra meeting with a case manager of approximately 1 hour. If this cannot be combined with a regular appointment, your travel expenses will be reimbursed. You may also be asked to wear an activity tracker (a kind of wristwatch) on your wrist for 6.5 months, at least 5 days a week (2 weeks before the operation up to and including 6 months after the operation).

All these matters have been described above under points 4, 5 and 6.

**8. If you do not want to participate or want to stop the study**

You decide if you want to participate in the study. Participation is voluntary. If you do not want to participate, you will be treated in the usual way for your knee arthroplasty by your hospital or clinic. This has no negative effect on the usual care. Your orthopaedic surgeon can tell you more about the treatment options available and their advantages and disadvantages.

If you do participate, you can always change your mind and stop, even during the study. You will then be treated in the usual way for your arthroplasty by your hospital or clinic. You do not have to say why you are quitting. However, you must immediately report this to the researcher. The data collected up to that point will be used for the research, unless you do not want this. Stopping the study will not harm your recovery.

If there is new information about the study that is important to you, the researcher will let you know. You will then be asked if you want to continue participating.

**9. End of the investigation**

Your participation in the study ends if:

• all questionnaires have been completed according to the schedule and the end of the entire study has thus been reached – this is 1 year after your surgery;

• you choose to stop;

• you appear to be or become pregnant and this will affect your return to daily (work) activities – this will be discussed with the researcher;

• the researcher thinks it is better for you to stop, for example due to serious complications of the surgery;

• Amsterdam UMC, your hospital or clinic, the government or the medical ethics review committee decides to stop the research.

After processing all the data, the researcher will of course also inform you about the most important results of the research. We expect the results in 2024. The researcher can then also tell you which care program you received and what the effect was on recovery. If you do not want to know this, you can tell the researcher. She can't tell you then.

**10. Use and storage of your data**

Your personal data will be used and stored for this research. This concerns data such as your name, address, date of birth and data about your health. The collection, use and storage of your data is necessary to answer the questions asked in this study and to publish the results. We ask for your permission for the use of your data.

*Confidentiality of your data*

To protect your privacy, your data is assigned to a code. Your name and other data that can directly identify you will be omitted. Your personal data can only be traced with the key to the code. The key to the code remains safely stored at the research location - the Amsterdam UMC, location VUmc. No individual patient data will be shared, only group averages. Your name or other data cannot be identified in these averages. The data in reports and publications about the research cannot be traced back to you either.

*Access your data for verification*

Only a limited number of researchers will have access to your data. Also to the data without code. This is necessary in order to check whether the research has been carried out properly and reliably. The researchers who have access to your data for verification purposes are: the committee that monitors the safety of the research and a monitor who works for the Amsterdam UMC. They keep your data secret. We ask you to give permission for this inspection.

*Data retention period*

Your data must be kept for 15 years at the research location – Amsterdam UMC, location VUmc.

*Storage and use of data for other research*

After this study, your data may also be important for other scientific research in the field of recovery and return to (work) activities after knee arthroplasty. Therefore, your data will be kept for 15 years. It is possible that the researchers would like to approach you again after this study for a follow-up study, for example to ask you an extra question that is important for this or a subsequent study. You can mark on the consent form (Appendix B) whether you consent to this. If you do not give permission for this, you can still participate in this research.

*Withdraw consent*

As mentioned, you can always withdraw your consent to the use of your personal data. This applies to this research as well as to storage and use for future research. The research data collected up to the moment you withdraw your consent will still be used in the research.

*Learn more about your rights when processing data*

For general information about your rights when processing your personal data, you can consult the website of the Dutch Data Protection Authority.

If you have any questions about your rights, please contact the person responsible for the processing of your personal data. For this study, that is: Amsterdam UMC, Location VUmc.

If you have any questions or complaints about the processing of your personal data, we recommend that you first contact the Amsterdam UMC, location VUmc. You can also contact the Data Protection Officer of the institution or the Dutch Data Protection Authority.

*Registration of the research*

Information about this research is also included in an overview of medical-scientific research, namely the CCMO register (https://www.ccmo.nl/ - click on 'CCMO-register'). It does not contain any data that can be traced back to you. After the research, the website can show a summary of the results of this research. You will find this study under the title “The (cost) effectiveness of a transmural intervention program for knee arthroplasty patients in the working population”.

**11. Subject Insurance**

Insurance has been arranged for everyone who participates in this study. The insurance covers damage caused by the study. Not all damage is covered. The insurance form provides more information about the insurance and the exceptions. It also states who you can report the damage to.

**12. Inform the treating specialist**

We always send your treating orthopaedic surgeon an e-mail to let you know that you are participating in the study. We will also inform your general practitioner. This is for your own safety. If you do not agree with this, you cannot participate in this study.

**13. No Compensation for Participation**

The care program that you receive during the study costs you nothing. You will not be paid for participation. The possible appointment with your case manager may require travel costs. Travel and parking costs needed for participation in this study will be reimbursed.

**14. Any questions?**

If you have any questions, please contact the executive researcher. For independent advice about participating in this study, please contact the independent doctor. He knows a lot about the study, but has nothing to do with this investigation.

If you have any complaints about the study, you can discuss this with the researcher or your treating orthopaedic surgeon. If you prefer not to, you can contact the complaints officer.

**15. Signing consent form**

We hope you have had enough information and time to decide if you want to participate in this study. If you want to participate, we would like to receive your written confirmation with your name and signature. With this permission you indicate that you have understood the information and agree to participate in the study.

Both you and the researcher will receive a signed version of this consent form.

Thank you again for considering participating in this study!

**Informed Consent Form**

- I have read the information letter. I could also ask questions. My questions have been answered satisfactorily. I had enough time to decide whether to participate.
- I know that participation is voluntary. I also know that I can decide at any time not to participate or to stop the study. I do not have to give a reason for that.
- I give permission to inform my orthopedic surgeon, general practitioner and coordinating researcher of my hospital or clinic that I am participating in this study.
- I give permission for the collection and use of my data to answer the research question in this study.
- I know that only specifically appointed researchers can access my data for the purpose of monitoring the research. These people are listed in this information letter. I consent to such access by these persons.
- I know that if I become pregnant, I must inform the researcher immediately and may no longer be able to participate in the study.
- I consent to the retention period of 15 years of my personal data.

- I do

□ want to be informed about which treatment I had/to which group I was assigned

□ not want to be informed about which treatment I had/to which group I was assigned

- I

□ do give permission to approach me again after this study for follow-up

□ do not give permission to approach me again after this study for follow-up

- I want to participate in this research.

Name:

Signature:

Date : __ / __ / __
